# Similar Strains of Coagulase-Negative Staphylococci Found in the Gastrointestinal Tract and Bloodstream of Bacteremic Neonates

**DOI:** 10.1155/2020/3509676

**Published:** 2020-07-21

**Authors:** Jennifer O. Adeghate, Emese Juhász, Miklós Á. Iván, Júlia Pongrácz, Katalin Kristóf

**Affiliations:** ^1^Institute of Laboratory Medicine, Semmelweis University, Budapest 1082, Hungary; ^2^Department of Ophthalmology, University of Pittsburgh, Pittsburgh, PA 15213, USA

## Abstract

**Objectives:**

Premature neonates are susceptible to opportunistic and nosocomial infections. Efforts have been made to determine whether the neonatal gut microbiome possesses potential for causing bloodstream infections in newborns via microbial translocation from the gastrointestinal tract. We aimed to examine similarities in coagulase-negative staphylococci (CoNS) strains found in the gastrointestinal tract and bloodstream in bacteremic neonates.

**Methods:**

CoNS strains isolated from blood cultures and perianal and pharyngeal swab samples of neonates from two neonatal intensive care units were investigated using matrix-assisted laser desorption/ionization time-of-flight mass spectrometry and pulsed-field gel electrophoresis. Molecular mass and genetic similarities of CoNS strains were compared.

**Results:**

Marked similarity was found in the molecular mass and genetic profile of examined CoNS isolates from blood cultures and perianal/pharyngeal samples. The percentage of neonates developing bacteremia following perianal and pharyngeal colonization by CoNS was significantly higher when compared to those colonized by Enterobacteriales species (*p* < 0.0002).

**Conclusions:**

CoNS colonizing the gut may be a source of bacteremia in neonates. Enterobacteriales species do not contribute as significantly to bacteremia when compared to CoNS, and may be protective against gut mucosa-originated systemic infection.

## 1. Introduction

Premature neonates are a patient population that is susceptible to opportunistic and nosocomial infections, particularly in the setting of their undeveloped immune systems [1]. Both full-term and premature neonates treated in neonatal intensive care units (NICU) are at a high risk of acquiring invasive infections due to their need for a complex level of care [2]. Over the past few years, more emphasis has been placed on discovering the components of the neonatal gut microbiota [3, 4], in search of a better understanding of the development of bloodstream infections (BSIs) in this patient population.

Several factors are involved in the gut colonization process including (a) the mode of delivery of the neonate, (b) the type of nutrition administered after birth, and (c) whether or not the neonate has been exposed to larger doses of antimicrobial drugs [5]. The first set of microorganisms to colonize the gastrointestinal (GI) tract will eventually determine the features of the gut microbiota later in life. If the original microbiota does not develop the necessary components or does not develop at the right pace—a process called “dysbiosis” [6]—then this will affect the neonate later on by increasing the likelihood of developing various autoimmune or atopic diseases, such as asthma and other allergic conditions [7]. Additionally, the overuse of antibiotics kills the bacteria that would otherwise colonize and protect the GI tract, leaving the gut of the neonate less populated by protective bacteria in a form of the so-called “hygiene hypothesis” [8]. Other conditions that may develop later in life include Crohn's disease in neonates with extreme antibiotic exposure [9] and celiac disease in neonates delivered via Cesarean section [10].

Studies have shown that the GALT (gut-associated lymphoid tissue) is gradually desensitized to microorganisms within the gut due to crosstalk between the GALT and the mucosa [7, 11]. Weng et al. showed that this is an inflammatory process initiated by colonizing bacteria, which produces signals that activate the GALT and the immune system. Stimuli include reactive oxygen species and pathogen-associated molecular patterns [6, 7]. These communications allow for the gut to develop tolerance to the colonizing gut microbiota and to alimentary antigens [6]. The process by which the GI tract develops tolerance to the colonizing commensal gut microbiota is thought to occur via production of IgA immunoglobulins against their antigens [12]. This process is complemented by IgA immunoglobulins obtained exogenously from breast milk [13, 14].

Obligate anaerobes have been shown to play an important role in the protection of the GI mucosa; therefore, their absence in the premature gut poses a great threat to its integrity by decreasing the effectiveness of the epithelial barrier [15, 16]. This causes the otherwise harmless colonizing microbiota of the GI tract, or in some cases pathogenic microorganisms acquired from the environment, to translocate through the mucosa, reach the blood vessels within the intestinal wall, and disseminate throughout the body [17]. In healthy, developed infants, the likelihood of bacteremia arising after such processes is low due to sufficient eradication of the microorganisms by cellular and humoral mechanisms of the immune system. However, since immunity to infection is low in premature neonates, manifest septic states can occur more easily.

Bloodstream infections (BSIs) are the most common form of nosocomial disease in all neonates [18]. In general, BSIs are becoming more prevalent due to the increased use of invasive medical devices and the overall lengthening of hospital stays. Because of these factors, neonates treated in the ICU setting are particularly susceptible to acquiring nosocomial infections. As such, the length of hospitalization has been shown to correlate with the prevalence of late-onset BSIs in very-low birth weight neonates [19, 20]. It has been shown that almost 40% of all neonates, both full-term and premature, being treated in the NICU will contract a nosocomial infection [21]. This number is much higher (67%) in developing countries due to differences in the standards of healthcare, such as hygiene regulations and practices [22]. The mortality rate in neonates affected by invasive nosocomial infections depends highly on gestational age and weight, length of hospital stay, and the type of pathogen involved in a bloodstream infection. Early-onset BSIs are known to have a higher mortality rate than late-onset BSIs due to the involvement and ultimate failure of multiple organs [23].

Most coagulase-negative staphylococci (CoNS) are found on the skin and mucosal surfaces, and are implicated in a variety of opportunistic and nosocomial infections, with *S. epidermidis* being the most common (about 70–80% of infections) [24]. CoNS possess a strong potential for causing invasive BSIs in newborns, especially in those born prematurely. They have been shown to be the most common cause of bacteremia in neonates, causing more than 50% of bloodstream infections (BSIs) in this group of patients [25–27]. This was also shown by Shivanna et al. [28], where 66% of isolates from positive blood cultures of neonates with BSIs were CoNS. Mortality due to suspected BSI caused by CoNS has been shown to occur in up to 10% of neonates treated in the NICU [29, 30]. The process of BSIs is thought to be associated with translocation of these microorganisms from the gastrointestinal (GI) tract into the bloodstream [24]. However, there is still scarce evidence as to whether the gut-colonizing CoNS may be involved directly in bloodstream infections by bypassing the mucosal immune system and entering the circulation. The most definitive and effective method for detecting CoNS bacteremia is by blood culture [2]. Surveillance samples are used to compare the strains isolated from invasive samples to those residing in various organ systems [31]. This method of identification is widely used in NICUs in order to monitor the composition of the commensal gut microbiota.

Virulence factors associated with CoNS are involved mainly in the formation of biofilms around their colonies on the surface of inanimate objects such as intravenous catheters and endotracheal tubes [32]. Within this biofilm layer, they are unreachable to the components of the cellular or humoral immune system, as well as to antibiotics [33–35], preventing them from being eliminated from the surfaces they are attached to [36]. They also produce various extracellular enzymes, including oxidases, hyaluronidases, lipases, hemolysins, and proteases, which protect them and aid in multiplication and survival. The severity of a CoNS infection depends highly on the virulence of the infectious strains [37], though in general, this group causes milder symptoms than coagulase-positive staphylococci such as *Staphylococcus aureus*. Also, when affecting neonates, prematurity is a significant risk factor in the development and progression of CoNS-related BSIs, with full-term neonates being more tolerant and resistant to major manifestations of these infections [37].

Coagulase-negative staphylococci have also been isolated from the stool of neonates with BSIs in a gene sequencing study completed by Madan et al. [38], showing that CoNS may in fact be predominantly present in the GI tract of premature neonates and may have a role in the development of sepsis. Although extensive research has been performed on the development of sepsis due to the translocation of Gram-negative bacteria from the gut into the bloodstream [39–41], more evidence is required to prove that CoNS could follow this route of infection as well.

Our aim was to determine whether CoNS strains colonizing the gut were related to strains isolated from blood cultures in bacteremic neonates. Further aims of this study were to analyze whether or not bloodstream infections occurring in the neonatal patient population could have been associated with resident gut bacteria and to determine the extent of protection that the resident gut bacteria provides when facing BSIs. This may provide further insight into whether CoNS colonizing the GI tract mucosa may be involved in systemic infection.

## 2. Materials and Methods

Our study included a three-year (January 2013 to December 2015) retrospective analysis of 1118 neonates hospitalized in the NICUs of two large university teaching hospitals in Budapest, Hungary. The microbiological results of clinical samples, such as blood cultures and peritoneal fluid samples (invasive sampling), and perianal and pharyngeal swab samples (surveillance sampling), were examined. From these 1118 neonates, a total of 5093 perianal samples (average 4.6 samples per neonate), 4022 pharyngeal surveillance samples (average 3.6 samples per neonate), and 4294 blood cultures (average 3.8 samples per neonate) were obtained. Of the 4294 blood cultures obtained, 588 blood cultures were positive for microorganisms. Of these, 449 blood cultures were positive for CoNS and 45 for Enterobacteriales species; however, only 390 CoNS-positive and 43 Enterobacteriales species-positive blood cultures had concurrent surveillance samples (perianal and pharyngeal swabs) obtained for genetic analysis and comparison of strains. Multiple samples were taken per neonate, as they were followed over time during the course of their suspected infection. Samples may also have been insufficient or contaminated and therefore had to be repeated. Since multiple surveillance samples and blood cultures were collected from each newborn, the results were depicted by sample, not by the number of neonates. Our prospective analysis of species identification and antibiotic susceptibility testing were performed on each blood culture isolate, but perianal and pharyngeal isolates were identified only as CoNS or Enterobacteriales. Bacteria cultured from perianal swab samples were accepted as representative for predominant gut-colonizing bacteria.

Neonates whose blood cultures were positive for either of the two most significant neonatal CoNS pathogens (*S. epidermidis* and *S. haemolyticus*) were further investigated. In a prospective ten-month period (September 2015 to June 2016), *S. epidermidis* and *S. haemolyticus* isolates cultured from both invasive and surveillance samples were collected and stored in glycerol-supplemented broth at −20°C for later use. Only perianal and pharyngeal isolates that were collected during this ten-month period were identified at species levels. Otherwise, perianal and pharyngeal isolates were identified as CoNS or Enterobacteriales and further protein mass spectrometry and genetic analysis were not performed. The genetic diversity of these isolates was compared using matrix-assisted laser desorption/ionization time-of-flight mass spectrometry (MALDI-TOF MS) and pulsed-field gel electrophoresis (PFGE).

MALDI-TOF MS analysis (Bruker, Daltonik GmbH, Fahrenheitstraße 4, Bremen, Germany) was used to identify bacteria during the routine diagnostic process. To reveal potential relatedness of bacterial isolates from different samples, MALDI-TOF MS analysis was performed on five *S. epidermis* and seven *S. haemolyticus* isolates selected at random from CoNS-positive surveillance and blood culture samples of two affected neonates. The direct smear method was used. Bacterial smears were pretreated with 70% formic acid and then overlaid by alpha-cyano-4-hydroxycinnamic acid matrix substance. The molecular mass of the proteins was compared based on spectrometric results using the FlexAnalysis program (Bruker). The mass spectrum of the proteins was subtracted from the baseline and smoothed once before analysis. The analysis was performed according to the Bruker FlexControl program. Although MALDI-TOF MS is an accepted form of relatedness testing, a more standardized method was required to characterize and compare the different isolates in this study. Therefore, we used PFGE to determine the genetic relativity of the isolates.

The genetic profiles of the previously described five *S. epidermidis* and seven *S. haemolyticus* isolates were obtained after SmaI DNA digestion. These were compared to the patterns from random control samples in order to confirm validity of the PFGE results. Pulsed-field gel electrophoresis was conducted based on a departmental protocol modified for CoNS [42]. All strains were grown overnight on blood agar plates at 37°C, and DNA was released from the cells using lysostaphin (Sigma, St. Louis, Missouri, USA). DNA was digested with SmaI (Promega, Madison, Wisconsin, USA) at 25°C for 3 hours. Gel electrophoresis was then performed in 1% agarose gel (Bio-Rad, Hercules, California, USA) using the CHEF-DR-II apparatus (Bio-Rad) in TBE buffer (1x Tris-borate-EDTA, pH: 8.3; Bio-Rad). Lambda DNA PFGE Marker (BioLabs, Budapest, Hungary) was used as a size standard in the first lane on each gel. Electrophoresis conditions were 14°C for 21 hours, with pulse time ranging from 5 to 60 seconds at an angle of 120°. Voltage was 6 V/cm. Gels were stained with ethidium bromide solution (Sigma) and digitalized with UVItec (Pharmacia Biotech, Piscataway, New Jersey, USA). The DNA band patterns obtained were analyzed visually with Diversity Database software, version 2.2.0 (Bio-Rad). Dendrograms were created using UPGMA clustering of a similarity matrix based on band-matching Dice coefficients (tolerance 1%, optimization 1%). Isolates showing indistinguishable or closely related band patterns (≤6 band differences, >8% similarity) were regarded as likely to be clonally related, as suggested by Tenover et al. [43].

## 3. Results

### 3.1. Bacterial Components of Perianal, Pharyngeal, and Blood Culture Samples

**Figure 1:**
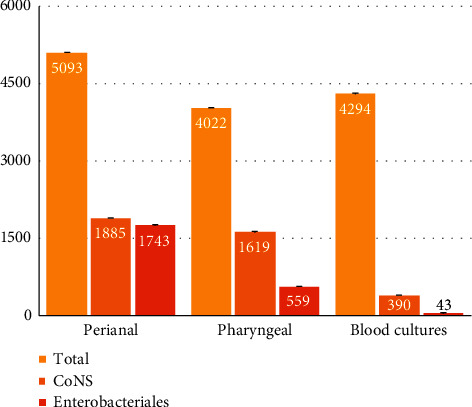
Total number of perianal, pharyngeal, and blood culture samples collected over 3 consecutive years (first column of each group). Of these, the number of cultures positive for CoNS and Enterobacteriales species is depicted in the second and third columns of each group, respectively. From 1118 neonates, a total of 5093 perianal samples, 4022 pharyngeal surveillance samples, and 4294 blood cultures were obtained. CoNS were isolated from 1885 (37%) of the perianal samples and 1619 (40.3%) of the pharyngeal samples. Enterobacteriales species were isolated from 1743 (34.2%) perianal samples and 559 (13.9%) pharyngeal samples. Of the 1885 CoNS-positive perianal samples and 1619 CoNS-positive pharyngeal samples, 216 and 174 blood cultures were positive for CoNS, respectively (total 390). Similarly, of the 1743 Enterobacteriales species-positive perianal samples and 559 Enterobacteriales species-positive pharyngeal samples, 34 and 9 blood cultures were also positive for Enterobacteriales species, respectively (total 43).

CoNS were isolated from 1885 (37%) of the perianal samples and 1619 (40.3%) of the pharyngeal samples. Enterobacteriales species were isolated from 1743 (34.2%) perianal samples and 559 (13.9%) pharyngeal samples (Figure 1). Of the neonates whose perianal and pharyngeal samples were positive for CoNS and Enterobacteriales species, the number of blood cultures positive for the same microbes was also determined. Of the neonates whose perianal samples were CoNS-positive (*n* = 1885), 216 blood cultures were also found to be positive for CoNS (11.5%). Meanwhile, of the neonates with CoNS-positive pharyngeal samples (*n* = 1619), 174 blood cultures were positive for CoNS (10.7%). Similarly, in the cohort with 1743 Enterobacteriales species-positive perianal samples, 34 (2.0%) blood cultures were also positive for Enterobacteriales species. In addition, out of 559 Enterobacteriales species-positive pharyngeal samples, 9 (1.6%) blood cultures were positive for Enterobacteriales species. The percentage distribution of neonates who acquired bacteremia following colonization by CoNS was markedly higher compared to bacteremia after colonization by Enterobacteriales species (*p* < 0.0002) (Table 1).

### 3.2. Distribution of Pathogens in All Positive Blood Cultures

**Figure 2:**
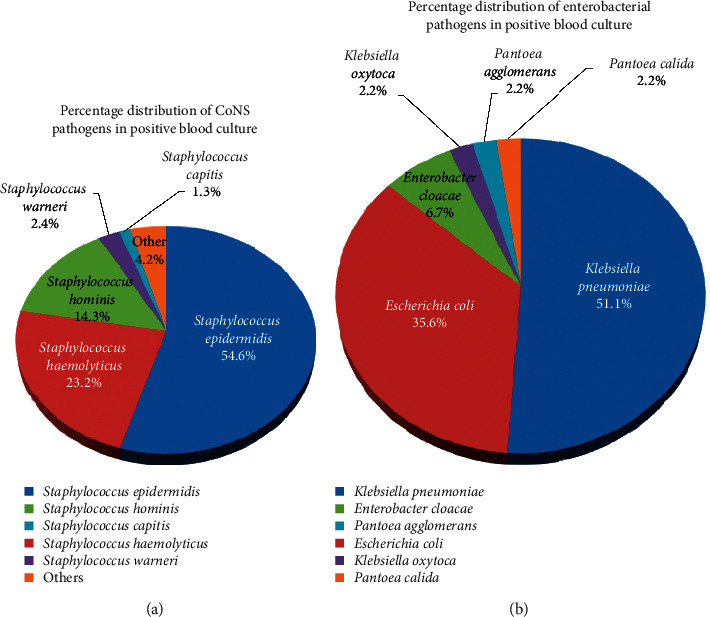
The percentage distribution of CoNS species (a) and Enterobacteriales species (b) in positive blood cultures of neonates.

There were a total of 588 blood cultures positive for microorganisms. Of these, 76.4% (*n* = 449) of isolates were CoNS species. The distribution of these species was as follows: *S. epidermidis* (*n* = 245; 54.6%), *S. haemolyticus* (*n* = 104, 23.2%), *S. hominis* (*n* = 64; 14.3%), *S. warneri* (*n* = 11; 2.4%), and *S. capitis* (*n* = 6; 1.3%). Other strains made up the remaining 4.2% (*n* = 19) (Figure 2(a)). Enterobacteriales species made up 7.7% (*n* = 45) of strains isolated from the 588 positive blood cultures. The majority of bacteria that comprised this group were *Klebsiella pneumoniae* (*n* = 23; 51.1%) and *Escherichia coli* (*n* = 16; 35.6%) strains. Others include *Enterobacter cloacae* (*n* = 3; 6.7%), *Klebsiella oxytoca* (*n* = 1; 2.2%), *Pantoea agglomerans* (*n* = 1; 2.2%), and *Pantoea calida* (*n* = 1, 2.2%) (Figure 2(b)).

### 3.3. Characterization of Pathogens by MALDI-TOF MS and PFGE

**Figure 3:**
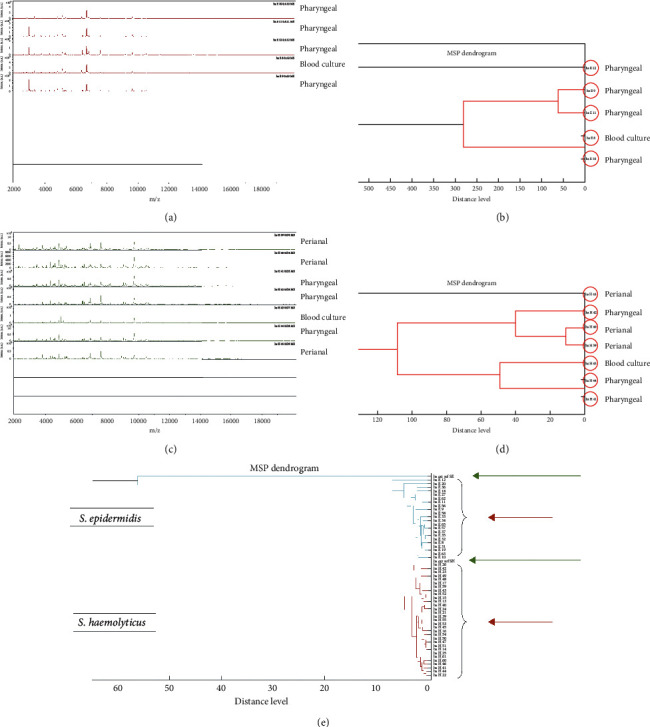
MALDI-TOF MS analysis (a) and representative dendrogram (b) of *S. epidermidis* strains isolated from a positive blood culture and 4 pharyngeal samples in a symptomatic neonate (*n* = 1). MALDI-TOF MS analysis (c) and representative dendrogram (d) of *S. haemolyticus* strains isolated from a positive blood culture, 3 pharyngeal samples, and 3 perianal samples in a symptomatic neonate (*n* = 1). Note the similarity in the graphs and dendrograms comparing pharyngeal, perianal, and blood culture strains (a–d). Main spectra dendrogram (e) of mass spectrum profiles of 23 *S. epidermidis* and 31 *S. haemolyticus* strains isolated from positive blood cultures, pharyngeal samples, and perianal samples from symptomatic neonates (short red arrows) was compared to 1 *S. epidermidis* and 1 *S. haemolyticus* strain from healthy neonates (long green arrows). In (a) and (c), the *x*-axis depicts the mass-to-charge ratio in m/z, while the *y*-axis depicts the intensity of the spectra in arbitrary units (a.u.).

MALDI-TOF MS analysis, including dendrograms of CoNS strains isolated from positive blood cultures, showed marked similarity to the strains isolated from the surveillance samples (Figures 3(a)–3(d)). The molecular mass of proteins belonging to *S. epidermidis* and *S. haemolyticus* strains isolated from blood cultures showed notable resemblance to the proteins of strains isolated from pharyngeal and perianal samples. The strains causing bacteremia had molecular masses that were different from those belonging to strains isolated from a healthy neonate (Figure 3(e)), indicating a possible difference in virulence between pathogenic and nonpathogenic strains of colonizing CoNS.

**Figure 4:**
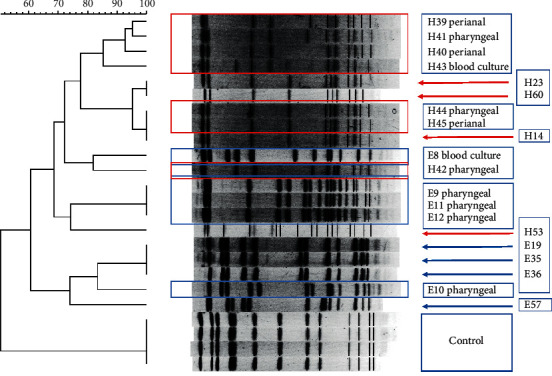
PFGE dendrogram of 5 *S. epidermidis* and 7 *S. haemolyticus* strains isolated from positive blood cultures, pharyngeal samples, or perianal samples from two symptomatic neonates, and 4 *S. epidermidis* and 4 *S. haemolyticus* strains isolated from healthy neonates (control). One random specimen was used as a control for testing the validity of the PFGE results.

The PFGE dendrogram and patterns of tested isolates are shown in Figure 4. Five *S. epidermidis* strains isolated from pharyngeal and perianal samples from a bacteremic neonate appeared to have a different genetic profile compared to that isolated from blood culture. Four *S. epidermidis* strains from four healthy neonates were used as controls. On the other hand, seven *S. haemolyticus* strains from pharyngeal, perianal, and blood culture samples from another bacteremic neonate appeared to have an identical genetic profile. Four *S. haemolyticus* strains from four healthy neonates were used as controls. Isolates from the healthy neonates were similar, but not identical. The dendrograms resulting from the PFGE and MALDI-TOF MS analysis showed marked resemblance.

## 4. Discussion

In our study, CoNS and Enterobacteriales strains isolated from blood and surveillance cultures of 1118 neonates were analyzed, and selected CoNS isolates were compared using MALDI-TOF MS and PFGE.

Several studies have shown the involvement of CoNS in the development of neonatal sepsis, as newborns treated in the NICU are frequently subjected to invasive and semi-invasive procedures that have a high potential for introducing bacteria into the bloodstream [28]. Coagulase-negative staphylococci comprised over 75% of microorganisms isolated from positive blood cultures in the neonates examined in our study, which corroborates with that of multiple studies which have shown that 50–66% of isolates from bacteremic neonates' blood cultures were CoNS [25–28].

Despite extensive research on the pathomechanisms by which GI-colonizing bacteria may cause systemic infection via translocation through the GI mucosa, the role of CoNS in this process, as a component of the gut microbiota, is unclear. We observed that despite the large number of enterobacteria colonizing the GI tract, only a small percentage of these microorganisms caused BSIs (1.6–2.0%) in the studied neonates, whereas among the CoNS-colonized neonates, 10.7–11.5% of CoNS caused blood stream infections. This implies that the infectivity of CoNS had surpassed that of other colonizing microbes such as Enterobacteriales species. Whether or not the source of CoNS bacteremia was the colonized gut mucosa raises the point that more attention should be paid to bacteremia caused by CoNS in previously CoNS-colonized neonates. Additional virulence testing would be helpful in determining whether specific strains of gut-colonizing CoNS are more likely than others to cause BSIs during the perinatal period. Further research is need to elucidate the combination of host factors and CoNS virulence factors that together could result in systemic infection from the naturally colonized gut mucosa. Especially in terms of prevention, further investigations are essential.

The molecular mass of proteins of *S. epidermidis* and *S. haemolyticus* strains isolated from selected neonates' blood cultures showed marked similarity to those isolated from the same neonates' perianal and pharyngeal samples. Moreover, the genetic makeup of *S. haemolyticus* strains of a symptomatic neonate showed identical PFGE genotypes. We also observed that the molecular mass and genome of these isolates in symptomatic neonates were different from those isolated from healthy neonates. This indicates that the molecular mass and genome of *S. epidermidis* and *S. haemolyticus* isolates from symptomatic neonates belonged to one genetic variant, while those obtained from the healthy neonate belonged to another. This may imply that isolates from respective invasive and surveillance samples were genetically related to each other in bacteremic neonates. It is also possible that the strains causing invasive infection may be from the same genetic variant indicating possible affinity for colonizing strains to cause systemic infection. More research is required in order to determine causality in these cases.

Our results showed that there was a much larger incidence of bacteremia in neonates whose GI tract was colonized by CoNS alone than in those colonized by Enterobacteriales species. The CoNS isolates representative for gut-colonizing bacteria matched the isolates cultured from blood samples, indicating that the bacteremia could have originated from the gut mucosa. This could indicate that either CoNS has a greater potential for becoming invasive from a colonizer state, or that the predominantly enterobacterial gut colonization plays more of a protective than infective role. In fact, it has been shown that the gut microbiota may indeed play a protective role in the maintenance of GI homeostasis [44].

Due to the severe consequences of bloodstream infections and an increase in antibiotic resistance [45], further insight into their pathogenesis should be sought in order to establish and implement preventive measures against systemic infection, particularly in the case of seemingly harmless colonizing CoNS.

## Figures and Tables

**Table 1:** Distribution of coagulase-negative staphylococci (CoNS) and enterobacterial bacteremia based on positive perianal and pharyngeal surveillance cultures over 3 consecutive years (%) (*p* < 0.0002).

Year	Type of culture (CoNS)	CoNS bacteremia (%)	Enterobacterial bacteremia (%)	Type of culture (Enterobacteria)
2013	Perianal (*n* = 608)	7.9	1.1	Perianal (*n* = 379)
	Pharyngeal (*n* = 475)	8.2	0.0	Pharyngeal (*n* = 125)
2014	Perianal (*n* = 635)	11.0	1.8	Perianal (*n* = 501)
	Pharyngeal (*n* = 548)	10.0	0.0	Pharyngeal (*n* = 145)
2015	Perianal (*n* = 642)	15.3	2.4	Perianal (*n* = 863)
	Pharyngeal (*n* = 596)	13.4	3.1	Pharyngeal (*n* = 289)
	Total perianal: 1885	34.2	5.3	Total perianal: 1743
	Total pharyngeal: 1619	31.6	3.1	Total pharyngeal: 559

## Data Availability

The clinical data used to support the findings of this study are included within the article. Deidentified raw data used to support the findings of this study are available from the corresponding author upon request.
